# A map of *cis*-regulatory modules and constituent transcription factor binding sites in 80% of the mouse genome

**DOI:** 10.1186/s12864-022-08933-7

**Published:** 2022-10-19

**Authors:** Pengyu Ni, David Wilson, Zhengchang Su

**Affiliations:** Department of Bioinformatics and Genomics, the University of North Carolina at Charlotte, Charlotte, NC 28223 USA

**Keywords:** *Cis*-regulatory modules, Transcription factor binding sites, Mouse

## Abstract

**Background:**

Mouse is probably the most important model organism to study mammal biology and human diseases. A better understanding of the mouse genome will help understand the human genome, biology and diseases. However, despite the recent progress, the characterization of the regulatory sequences in the mouse genome is still far from complete, limiting its use to understand the regulatory sequences in the human genome.

**Results:**

Here, by integrating binding peaks in ~ 9,000 transcription factor (TF) ChIP-seq datasets that cover 79.9% of the mouse mappable genome using an efficient pipeline, we were able to partition these binding peak-covered genome regions into a *cis*-regulatory module (CRM) candidate (CRMC) set and a non-CRMC set. The CRMCs contain 912,197 putative CRMs and 38,554,729 TF binding sites (TFBSs) islands, covering 55.5% and 24.4% of the mappable genome, respectively. The CRMCs tend to be under strong evolutionary constraints, indicating that they are likely *cis*-regulatory; while the non-CRMCs are largely selectively neutral, indicating that they are unlikely *cis*-regulatory. Based on evolutionary profiles of the genome positions, we further estimated that 63.8% and 27.4% of the mouse genome might code for CRMs and TFBSs, respectively.

**Conclusions:**

Validation using experimental data suggests that at least most of the CRMCs are authentic. Thus, this unprecedentedly comprehensive map of CRMs and TFBSs can be a good resource to guide experimental studies of regulatory genomes in mice and humans.

**Supplementary Information:**

The online version contains supplementary material available at 10.1186/s12864-022-08933-7.

## Background

Mouse is probably the most widely used model organism to understand mammal biology and pathology of human diseases. Thus, it is not surprising that mouse is the first sequenced non-human mammal [1]. Conserved syntenies between the human and mouse genomes provide a powerful tool to understand functions of the human genome based on the known functions of the mouse orthologous sequences [1]. This homology-based approach plays a critical role in annotating the coding DNA sequences (CDSs) in the human genome. However, the power of such comparative genomics approach is hampered in annotating human *cis*-regulatory sequences due to the lack of a good understanding of mouse orthologous sequences and their less conservation nature compared with CDSs [2]. c*is*-regulatory sequences such as promoters, enhancers and silencers are also called *cis*-regulatory modules (CRMs). While promoters are located upstream of target genes, enhancers and silencers can be far away (up to millions base pairs) from target genes, and they regulate the transcription of target genes independently of their locations and orientation [3, 4]. A CRM is made of clusters of transcriptional factor (TF) binding sites (TFBSs) of the same and different cooperative TFs, with a length ranging from hundreds to thousands of base pairs [5]. A CRM carries out its transcriptional regulatory function through specific bindings of cognate TFs to the TFBSs that it harbors. CRMs play equally important roles as CDSs in development, homeostasis, responses to environmental changes and evolution of organisms [6]. Diversity of CRMs may play even more important roles in driving diverse complex traits in humans [7] and mice [8]. For example, genome-wide association studies (GWAS) in humans have found that most (90%) complex trait-associated single nucleotide variations (SNVs) reside in non-coding sequences [9, 10]. Many of these SNVs overlap and disrupt TFBSs [11], thereby affecting gene transcription [12–16], and ultimately complex traits and diseases. On the other hand, many SNVs are in linkage disequilibrium (LD) with nearby TFBSs, and thus may not necessarily causal [17–27]. Hence, a better understanding of the mouse CRMs will not only aid to understand various aspects of mouse biology and make it an even better model of human diseases, but also will facilitate annotating human CRMs and understanding human biology. For example, studies of CRMs in human cells or tissues can be complemented by manipulating the orthologous CRMs in transgenic mouse in vivo using knockout and knockin techniques [1, 28, 29].

In fact, great efforts have been made in the last decade to systematically annotate CRMs and constituent TFBSs in the mouse genome by the research community including the mouse ENCODE consortium [30–32] and individual groups worldwide using state-of-the-art techniques. Particularly, an enormous amount of data have been generated using ChIP-seq techniques to locate CRM function-related epigenetic marks [33, 34] and TF bindings [35] in the genomes of various mouse cell/tissue types. Numerous machine-learning methods [36–42] have been developed to simultaneously predict CRMs and their functional states using location data of multiple epigenetic marks including histone modifications such as H3K4me1 [43–45], H3K4me3 [46] and H3K27ac [47], and chromatin accessibility (CA) measured by DNase I hypersensitivity [33] and transposase accessibility [34]. Although conceptually attractive, these methods suffer quite high false discovery rates (FDRs) [40, 48–53] due probably to low the specificity of these epigenetic marks used [48–51, 54] and at the same time, they might miss many CRMs in the genome because these data are only available in a few cell types [50]. Moreover, these methods do not predict constituent TFBSs in the CRMs, notwithstanding it is mainly the TFBSs in a CRM that determine its functions [5, 55]. More recently, the ENCODE phase 3 consortium [30] predicted 339,815 candidate *cis*-regulatory elements (cCREs) in the mouse genome based on overlaps between millions of DNase I hypersensitivity sites [56], transposase accessible sites [34], active promoter histone mark H3K4me3 [57] peaks, active enhancer mark H3K27ac [58] peaks, and insulator mark CTCF[59] peaks, in a large number of mouse cell/tissue types. Nonetheless, the cCREs with an almost uniform length of 272 bp are likely only fragments of full-length CRMs, because the known mouse enhancers have a mean length about 2,400 bp [60]. Moreover, the cCREs make up of 3.4% of the mouse genome [30], they might be largely under predicted.

To overcome the limitations of the existing methods, we proposed a two-step approach to first predict a map of CRMs and their constituent TFBSs in the genome using all available TF ChIP-seq data in the organism, and then predict functional states of all the predicted CRMs in any cell/tissue type of the organism using few epigenetic marks from the very cell/tissue type [50]. We recently developed a new CRM predictor dePCRM2 [50] for the first step of our approach. dePCRM2 works by identifying closely located clusters of TFBSs in a genome through integrating all available thousands of TF ChIP-seq datasets in the organism [50]. Unlike the existing methods, we use TF ChIP-seq data instead of CA and histone modification data to predict the loci of CRMs and constituent TFBSs, because it has been shown that TF binding is a more reliable predictor of CRM loci than CA and histone marks [48]. Using dePCRM2, we have predicted a highly accurate and unprecedentedly complete map of CRMs and constituent TFBSs in the human genome using then available 6,092 human TF ChIP-seq datasets [50]. In this study, we applied dePCRM2 to 9,060 mouse TF ChIP-seq datasets, and predicted an unprecedentedly complete map of CRMs and constituent TFBSs in 79.9% of the mouse genome. Validation of the map using orthogonal evolutionary and experimental data suggests that our predictions are highly accurate. The map can be a good resource to guide experimental studies of the regulatory genomes of both mice and humans.

## Results

### The 1,000 bp binding peaks for cooperative TFs in different datasets have extensive overlaps

**Fig. 1:**
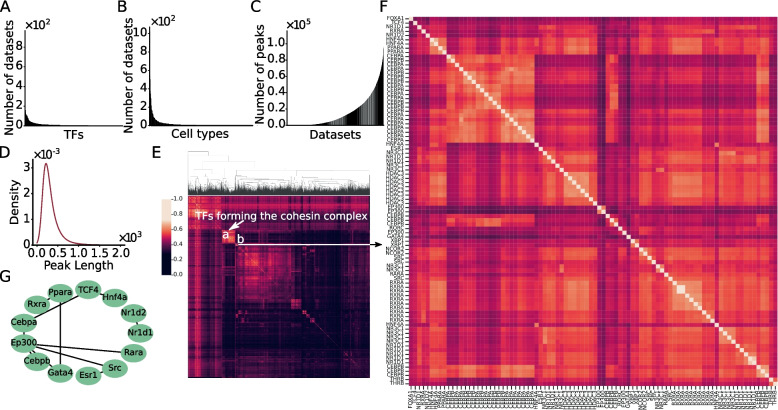
Evaluation of the TF ChIP-seq datasets. **A** Number of datasets collected in each cell/tissue types sorted in the descending order. **B** Number of datasets collected for each ChIP-ed TF sorted in the descending order. **C** Number of peaks in each dataset sorted in the ascending order. **D** Distribution of the lengths of originally called binding peaks in the 8,884 datasets. **E** Heatmap of overlaps of the 1,000 bp binding peaks between each pair of the datasets. The highlighted cluster *a* is formed by 50 datasets for Ctcf and varying number of datasets for its cooperative TFs Rad21, Stag1, and Smc1A in different cell/tissue types. **F** A blowup view of cluster *b* highlighted in E, formed by 88 datasets for 20 TFs. **G** Known physical interactions between 13 of the 20 TFs for which the 88 datasets form the cluster in **F**

After quality-control filtering of the 9,060 collected TF ChIP-seq datasets (Table S1) (Materials and Methods), we ended up with 8,884 datasets containing at least 20 binding peaks for 696 TFs in 435 cell line/tissue/organ types. As in the case in humans [50, 61], these datasets are high biased to a few well-studied cell/tissue types (Fig. 1A) and TFs (Fig. 1B). For example, 1,020, 504 and 545 datasets were collected from mouse embryonic stem cells, epithelial cells and macrophage in bone marrow, respectively, while only one dataset was generated from 68 cell/tissue types, including pancreas beta cell MIN6B1, superior cervical ganglion, hepatocellular carcinoma, and so on (Table S1). Moreover, 460 and 160 datasets were collected for TFs Ctcf and SpI1, respectively, while just one dataset was produced for 131 TFs, such as Tfcp2, Nelfb, Hoxd11, and so on (Table S1). The number of remaining binding peaks in a dataset vary widely, ranging from 20 to 110,347, with an average of 15,359 peaks (Fig. 1C). The length of the called binding peaks also vary widely, ranging from 21 to 11,047 bp with a mean of 315 bp, but the vast majority of them (98.7%) are shorter than 1,000 bp (Fig. 1D). We extracted 1,000 bp genomic sequences centered on the summits of the called binding peaks for motif-finding to identify motifs of both the ChIP-ed TFs and their cooperators in each dataset [50, 62]. Therefore, we extended the lengths of most (98.7%) of the originally called binding peak. We have previously shown that such extension (~ 1,000 bp) of called peaks does not affect finding the motifs of ChIP-ed TFs, which typically reside in the middle of the peaks, but allows to find motifs of cooperative TFs, which can reside anywhere along the extended peaks [50, 62].

In theory, the larger the number of TF ChIP-seq datasets available and used, and the less bias of the datasets to few TFs and cell/tissue types, the better predictions that dePCRM2 can achieve [50, 61]. To see whether such the highly biased datasets include enough datasets for cooperative TFs that are reused in different cell/tissue types, an assumption upon which dePCRM2 is designed for predicting CRMs and constituent TFBSs [50], we calculated an overlapping score ${S}_{o}$ (formula 1) for each pair of the 8,884 filtered datasets, and hierarchically clustered them. As show in Fig. 1E, there are numerous overlapping clusters among the datasets which are either for largely the same TFs that were ChIP-ed in different cell/tissue types, or for different known cooperative TFs that were ChIP-ed in the same and/or different cell/tissue types. For example, as seen in the human datasets [50], a cluster is formed by 50 datasets for cooperative TFs Ctcf, Rad21, Stag1, and Smc1A in various cell/tissue, reflecting the conserved cooperative relationships of the TFs in forming the cohesin complex [63]. Shown in Fig. 1F is another example of cluster formed by 88 datasets for 20 TFs in various cell/tissue types, many of these TFs are known or likely to collaborate with each other according their physical interactions documented in the BioGRID [64] and reactome [65] databases (Fig. 1G). Therefore, notwithstanding these datasets are highly biased to few TFs (Fig. 1A) and cell/tissue types (Fig. 1B), they include datasets of many cooperative TFs that are reused in various cell/tissue types. The 1,000 bp peaks in all the 8,884 datasets contain a total of 136,441,496,000 bp, which is 50.1 times the size of the mouse genome (version mm10/GRCm38), but cover only 2,178,603,271 bp (79.9%) of the mappable genome (2,725,521,370 bp). Compared with the originally called peaks that cover a total of 1,398,035,305 bp (51.3%) of the mappable genome, we substantially increased the coverage of the genome by extending the called peaks to 1,000 bp, the size of shorter enhancers [60]. dePCRM2 will predict which DNA segments in the 79.9% genome regions covered by the 1,000 bp peaks are CRM candidate (CRMCs), and which are non-CRMCs, based on cooccurring patterns of putative TFBSs of motifs found in the binding peaks in all the datasets.

### Most of identified unique motifs (UMs) resemble known motifs and show intensive cooccurring pattens

**Fig. 2:**
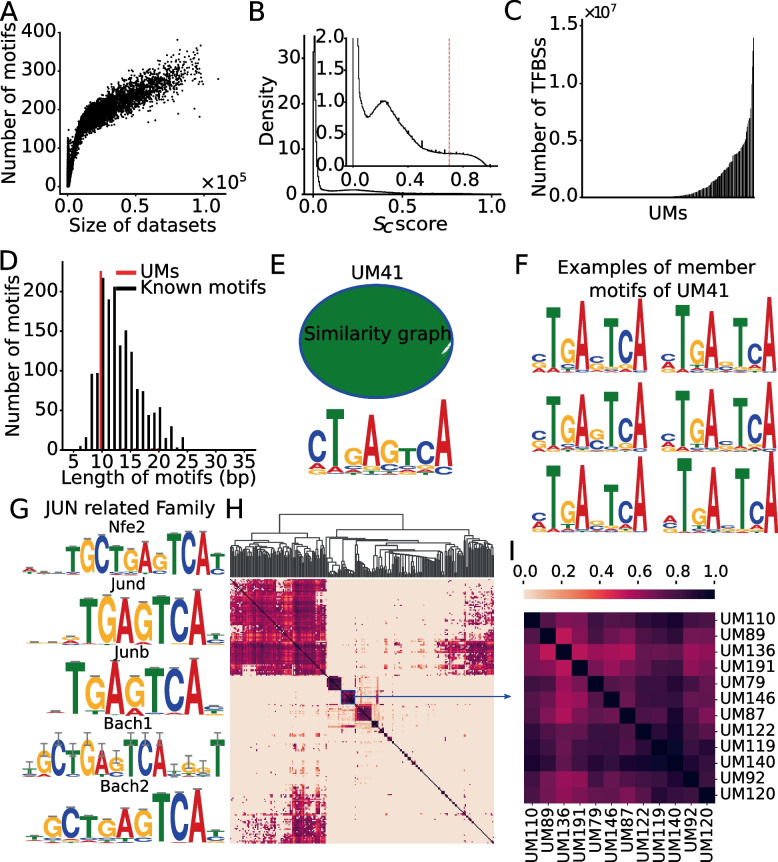
Prediction of UMs. **A** Relationship between the number of predicted motifs in a dataset and the size (the number of binding peaks in the dataset). The datasets are sorted in the ascending order of their sizes. **B** Distribution of cooccurrence scores (${S}_{c}$) of motif pairs found in each dataset. The dotted vertical line indicates the cutoff value of ${S}_{c}$ for predicting cooccurring pairs (CPs). **C** Number of putative binding sites in each of the UMs sorted in the ascending order. **D** Distribution of the lengths of the UMs and known motifs in the HOCOMOCO and JASPAR databases. **E** The motif similarity graph (upper panel) and the logo (bottom panel) of UM41 containing 11,799 member motifs. In the graph, the nodes (colored in blue, each representing a member motif) are arranged on the rim of the ova, and two member motifs are connected by an edge (colored in green) with SPIC score > 0.8. **F** Logos of six examples of highly similar member motifs of UM41. **G** UM41 matches known motifs of five TFs of the JUN-related family. **H** Heatmap of the cooccurrence/interaction networks of the 238 UMs, names of most UMs are omitted for clarity. **I** A blowup view of the indicated cluster in **H**, formed by 14 UMs, of which UM116, UM14, UM26, UM28, UM29, UM32, UM45, UM53, UM55, and UM57 match known motifs (see main text)

dePCRM2 [50] starts by identifying all possible motifs in each dataset using ProSampler, an ultrafast motif finder [62]. ProSampler finds at least one motif in 8,294 (93.4%) of the 8,884 datasets, with a total of 1,062,339 motifs found. As shown in Fig. 2A, the number of motifs found in a dataset increases with the number of peaks in it, but becomes stabilized around 250 when the number of peaks is above 50,000. dePCRM2 next identifies co-occurring motifs pair (CPs) as potential motifs, thereby filtering out most spurious motifs. To do so, dePCRM2 computes a co-occurring score ${S}_{c}$ (formula 2) for each pair of motifs in each dataset and selects the pairs with high scores as CPs. As in the case of human genome [50], the ${S}_{c}$ scores show a trimodal distribution (Fig. 2B). dePCRM2 selects motifs pairs as PCs that account for the mode with the highest ${S}_{c}$ scores (${S}_{c}$ > 0.7 by default). More specifically, dePCRM2 identifies 4,028,221 CPs containing 225,809 (21.3%) potential motifs from 7,076 (85.3%) of the 8,294 datasets, while filtering out the remaining 1,218 (15.7%) datasets where no CPs are kept, and 836,530 (78.7%) possible spurious motifs. Many motifs in different CPs can be sub-motifs of the same TF, or of different members of a TF family that recognize highly similar motifs [66, 67]. Therefore, dePCRM2 clusters the 225,809 motifs in the 4,028,221 CPs by constructing a graph whose nodes are the motifs and edges are the SPIC similarity score [68] between the motifs pairs, and then cutting the graph into dense subgraph as clusters of similar motifs. This results in 276 clusters, each containing from 28 to 49,308 motifs (Figure S1A). From these 276 motif clusters, dePCRM2 identifies 238 unique motifs (UMs) (Figure S1B). The UMs contain highly varying number of TFBSs, ranging from 72 to 14,025,382 with an average of 1,107,677 (Fig. 2C). The lengths of the UMs range from 10 to 20 bp with a mean of 10.3 bp, and are in the range of the lengths of known TF binding motifs (Fig. 2D). The bias of the lengths of UMs to 10 bp is due to the limitation of ProSampler that needs to be improved. As expected, the UMs and their member motifs are highly similar to one another. For example, the 11,799 member motifs of UM41 form a dense subgraph/cluster (Fig. 2E), and UM41 resembles its highly similar member motifs (Fig. 2E, F). To evaluate the UMs, we compared the 238 UMs against 875 annotated non-redundant motifs in the HOCOMOCO [69, 70] and JASPAR [71] databases using TOMTOM [72]. Of the 238 UMs, 146 (61.3%) match at least one annotated motif, and 113 (77.4%) of the 146 UMs match at least two (Table S2), suggesting that most of the UMs might represent the motifs of the same TF family/superfamily which bind highly similar motifs [66, 67]. For instance, UM41 matches known motifs of five TFs of the “Jun-related factors” family (Jund, Bach1, Bach2, Junb and Nfe2) (Fig. 2G), and five TFs of the “Fos-related factors” family (Atf3, Fosl2, Fosb, Fosl1 and Fos) (Table S2). On the other hand, the remaining 92 UMs might be novel motifs of unknown cognate TFs. We also evaluated the coverage of the UMs on motif families in the two databases [70, 71], and found that 82 (64.1%) of the 128 annotated TF motif families match one of the 238 UMs (Table S2), indicating that our predicted UMs recovery most of the known TF motif families.

To model cooccurring patterns of the UMs and interactions between their cognate TFs, dePCRM2 computes a cooccurrence/interaction score *SINTER *(formula 3) between each pair of UMs based on the co-occurrence of binding sites of UMs. As shown in Fig. 2H, there are extensive cooccurrences between the UMs and interactions of their cognate TFs. These patterns of cooccurrences of the UMs indeed reflect the interactions among their cognate TFs or TF families for transcriptional regulation. For example, in a cluster formed by 14 UMs (Fig. 2I), 10 of them (UM14, UM26, UM28, UM29, UM32, UM45, UM53, UM55, UM57 and UM116) match known motifs of TF families. More specifically, UM116 matches Msantd3, UM14 matches Ctcfl, UM26 matches Nfe2|Fosb|Atf3|Bach1|Pknox1|Jund|Nkx2-2|Jdp2|Fos|Junb|Fosl1|Fosl2|Batf|Msantd3|Bnc2|Mafk|Pbx3|Batf3|Jun, UM28 matches Zfp57|Atf3, UM29 matches Sp3|Mxi1|Nr1h4|Plagl1|Zfx|Klf3|Rfx1, and UM57 matches Nkx2-5|Fos|Fosb|Atf3|Pbx3|Junb|Jund|Pknox1|Fosl1|Batf3|Fosl2|Jun|Batf|Nkx2-2|Msantd3|Bnc2, etc. Some of these TFs are known collaborators in transcriptional regulation, such as Fos and Jun [73–76], Atf3 and Jun [77], Pbx3 and Pknox1 [78], Jun and Batf [78].

### Prediction of CRMs and constituent TFBSs in the mouse genome

**Fig. 3:**
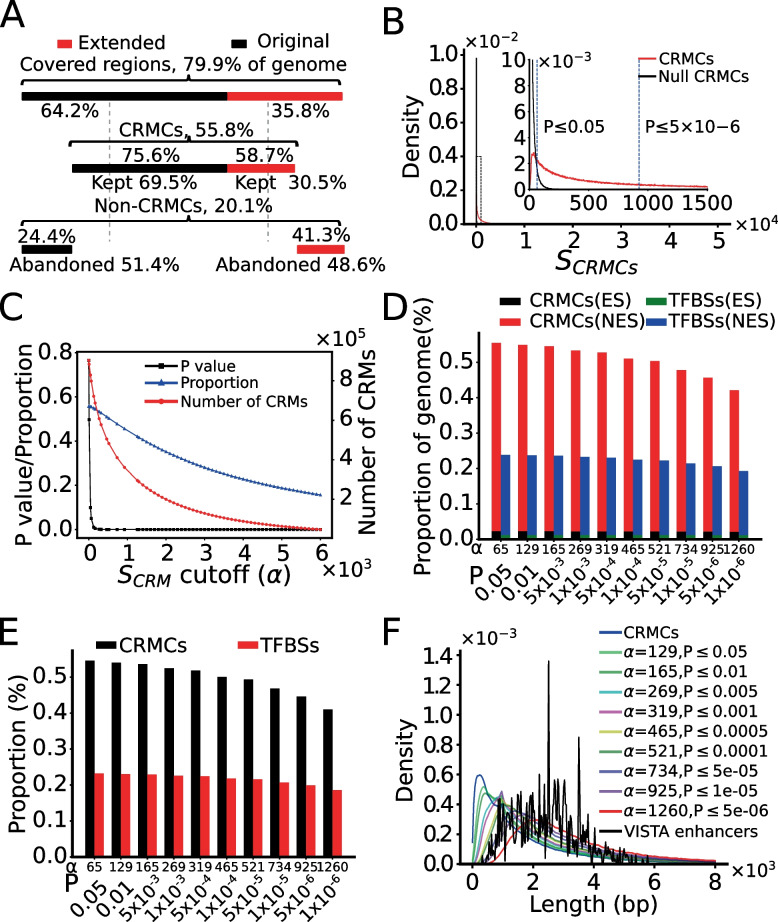
Prediction of CRMs using different ${S}_{CRM}$ cutoffs. **A** A cartoon shows the proportions of the 79.9% of genome regions covered by originally called binding peaks (64.2%) and their extended parts (35.8%) as well as their relative contributions to the predicted CRMs (kept original (69.5%) and kept extended (30.5%)) and non-CRMCs (abandoned original (51.4%) and abandoned extended (41.3%)). Percentage above the lines are the proportion of originally called binding peaks and their extended parts that are predicted to be CRMCs and non-CRMCs. **B** Distribution of the ${S}_{CRM}$ scores of the CRMCs and the Null CRMCs. The inset is a blowup view of the indicated regions. The dotted vertical lines indicate ${S}_{CRM}$ cutoffs for the corresponding *p*-values. **C** Number of the predicted CRMs, proportion of the genome predicted to be CRMs and the corresponding *p*-value as functions of the ${S}_{CRM}$ cutoff α. **D** Percentage of the genome that are predicted to be CRM and TFBS positions in exonic sequences (ESs) and non-exonic sequences (NESs) using various ${S}_{CRM}$ cutoffs and corresponding *p*-values. **E** Percentage of NESs that are predicted to be CRMs and TFBSs using various ${S}_{CRM}$ cutoffs and corresponding *p*-values. **F** Distribution of the lengths of CRMs predicted using different ${S}_{CRM}$  cutoffs and corresponding *p*-values

To predict CRMs and constituent TFBSs in the mouse genome, dePCRM2 projects the TFBSs of the UMs to the genome and links adjacent TFBSs if their distance is less than 300 bp (roughly, the length of two nucleosomes). dePCRM2 predicts each linked sequence as a CRM candidate (CRMC) and each sequence between two adjacent CRMCs in the peak-covered regions as a non-CRMC, thereby partitioning the peak-covered genome regions in two exclusive sets, CRMCs and non-CRMCs. Concretely, dePCTM2 predicts a total of 912,197 CRMCs and 1,270,937 non-CRMCs in the peak-covered genome regions, consisting of 55.5% and 24.4% of the genome, respectively. The CRMCs contains a total of 125,113,756 TFBSs, consisting of 23.9% of the genome and 42.9% of the CRMCs (Fig. 3A). Many of these TFBSs have overlaps due partially to the aforementioned limitation of our motif-finder ProSampler, although it has been shown that certain patterns of transcriptional regulation are achieved by competitive or cooperative binding of the same or different TFs to overlapping TFBSs in a CRM [79–83]. We connected each two adjacent overlapping putative TFBSs, resulting in a total of 38,554,729 non-overlapping putative TFBS islands with a mean length of 17 bp.

Interestingly, as in the case of human genome [50], 75.6% of genome positions of the originally called binding peaks were predicted as CRMC positions (kept-original), while the remaining 24.4% were predicted as non-CRMC position (abandoned-original) (Fig. 3A). On the other hand, 58.7% of the extended positions were predicted as CRMCs (kept-extended), while the remaining 41.3% were predicted as non-CRMC positions (abandoned-extended) (Fig. 3A). These results suggest that originally called binding peak positions may not necessarily parts of CRMs, while many flanking positions of the called peaks may be parts of CRMs. Therefore, as we concluded earlier [50], extension of the originally called peaks to roughly half of the mean length (1,000 bp) of known of CRMs (2,400 bp) [60] could greatly increase the chance of finding more CRMs in genomes.

To evaluate the CRMCs, dePCRM2 computes a ${S}_{CRM}$ score (formula 3) and a corresponding *p*-value for each CRMC (Materials and Methods). As shown in Fig. 3B, the distribution of the ${S}_{CRM}$ scores of the CRMCs is strongly right-skewed relative to that of the Null CRMCs with the same number and lengths of the CRMs (Materials and Methods), suggesting that the CRMCs are unlikely produced by chance. Moreover, with the increase in the ${S}_{CRM}$ cutoff α, the corresponding p value drops rapidly, while both the number of predicted CRMs with a ${S}_{CRM}>\mathrm{\alpha }$ and their coverage of the genome decrease only slowly (Fig. 3C), suggesting that most of the CRMCs have quite low *p*-values. More specifically, when the *p*-value drops precipitously from 0.05 to 1.00 × 10^–6^ the number of predicted CRMs and their coverage of the genome only decrease from 798,257 to 295,382, and from 55.5% to 42.1%, respectively (Fig. 3D). Moreover, with the *p*-value dropping from 0.05 to 1.00 × 10^–6^, the coverage of putative TFBSs on the genome decreases only from 23.9% to 19.3%, and their percentage in the CRMs increases only from 43.0% to 45.8% (*p*-value ≤ 1.00 × 10^–6^) (Fig. 3D). As expected, in the 0.05 ~ 1.00 × 10^–6^ range of *p*-value cutoffs, the vast majority of the predicted CRM positions (94.9 ~ 95.9%) and constituent TFBS positions (93.8 ~ 94.8%) are located in non-exonic sequences (Fig. 3D), converging 41.0 ~ 54.70% and 18.6 ~ 23.2% of their lengths, respectively (Fig. 3E). Interestingly, the remaining 4.1 ~ 5.1% of the predicted CRM positions and 5.2 ~ 6.2% of constituent TFBS positions are located in exonic sequences (Fig. 3D), a well-known phenomenon in mammal genome [84–100]. We will address these exonic CRMs and TFBS positions in great detail elsewhere.

We next compared the lengths of predicted CRMs at different ${S}_{CRM}$ cutoffs α with those of known mouse enhancers in the VISTA database [60]. As shown in Fig. 3F, the predicted CRMCs have a shorter mean length (1,682 bp) than the VISTA enhancers (2,432 bp). This is not surprising since most VISTA enhancers are involved in complex embryonic development and tend to be longer than other types of enhancers [101]. However, with the increase in the ${S}_{CRM}$ cutoff α, the distribution of the lengths of predicted CRMs shifts to right. Specifically, 252,349 (27.7%) of the 912,197 CRMCs were shorter than the shortest VISTA enhancer (330 bp), but they cover only 2.1% of total length of the CRMCs, suggesting that they are likely either short CRMs or components of full-length enhancers remained to be fully predicted using more TF ChIP-seq datasets in the future. The remaining 659,848 (72.3%) CRMCs that are longer than the shortest VISTA mouse enhancer (330 bp) consist of 97.9% of the total length of the CRMCs, and they are likely full-length CRMs. Thus, the vast majority (97.9%) of the CRMC positions are covered by predicted full-length CRMs. The predicted CRMs and constituent TFBSs are available at (https://cci-bioinfo.uncc.edu).

### Predicted CRMCs tend to be under strongly evolutionary constraints

**Fig. 4:**
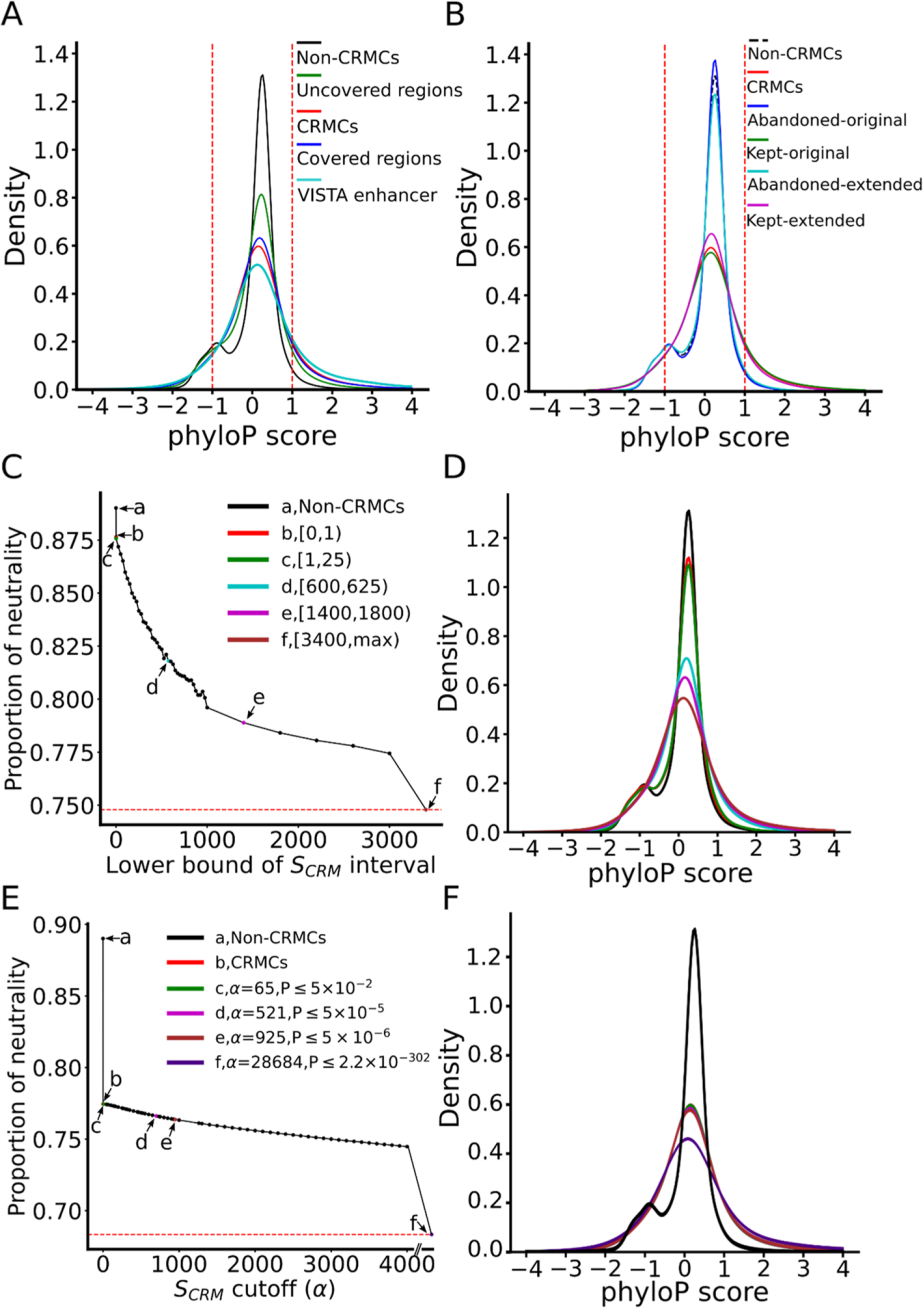
Different evolutionary constraints on the predicted CRMCs and the non-CRMCs in non-exonic sequences measured by phyloP scores. **A** Distributions of phyloP scores of nucleotide positions of the VISTA enhancers, the predicted CRMCs, the non-CRMCs, peak-covered regions and peak-uncovered regions. The area under the density curves in the score interval [-1, 1] is defined as the proportion of neutrality of the positions. **B** Distributions of phyloP scores the kept-original, the kept-extended, the abandoned-original and the abandoned-extended positions in comparison with those of the CRMCs and the non-CRMCs. The distributions for the kept-original positions and the kept-extended positions are significantly different from those of the abandoned-original positions and the abandoned-extended positions, respectively, *p* < 2.2 × 10^–302^ (K-S test). **C** Proportion of neutrality of the CRMCs with a ${S}_{CRM}$ score in different intervals in comparison with that of the non-CRMCs (a). **D** Distributions of the phyloP scores of the non-CRMCs and the CRMCs with ${S}_{CRM}$ scores in the intervals indicted by color and letters in (**C**). **E** Proportion of neutrality of the CRMs predicted using different ${S}_{CRM}$ score cutoffs and corresponding *p*-values in comparison with those of the non-CRMCs (a) and the CRMCs (b). **F** Distributions of the phyloP scores of the non-CRMCs, the CRMCs and the CRMs predicted using the ${S}_{CRM}$ score cutoffs and corresponding *p*-values indicated by color and letters in (**E**)

To see how the CRMCs and non-CRMC evolve, we plotted the distributions of the phyloP scores [102] of their nucleotide positions. The phyloP treats negative and positive selections in a unified manner and detects departures from the neutral rate of substitution in either direction, while allowing for clade-specific selection [102]. A positive phyloP score indicates the position is under purifying selection, a negative score indicates the position is under positive selection, and a score around zero means the position is selective neutral or nearly so. For convenience of discussion, we consider a position with a score in the range [-δ, δ] (δ > 0) to be selectively neutral, in the range (δ, max) to be under positive selection, and in the range (min, -δ) to be under negative selection, respectively. We define the proportion of neutrality of a set of position as the areas under the distribution of the scores within range [-δ, δ], and choose δ = 1 in this study. For this analysis, we focused on the CRMCs and the non-CRMCs in non-exonic sequences, because including exonic sequences would confound the analysis due to their coding functions. The distribution of the phyloP scores of the non-CRMCs peaks at the neutral range with a proportion of neutrality of 0.89 (Fig. 4A), suggesting that the non-CRMC positions are largely selective neutral as expected, although it is possible that some non-CRMC positions that are under some level of selections might have functions other than *cis-*regulatory. In contrast, the distribution of the phyloP scores of the CRMC positions displays a lower peak in the neutral range with a proportion of neutrality of 0.77 (Fig. 4A), and spreads to both negative selection and positive selection ranges. These results indicate that CRMC positions are more likely to be under evolutional constraints than the non-CRMC positions. Thus, the CRMCs are more likely to be functional than non-CRMCs, although some CRMC positions that are selected neutral might not be functional. Notably, the mouse VISTA enhancers are even more likely to be evolutionarily conserved than our predicted CRMCs (Fig. 4A), although the former are largely a small subset of the latter (see below). This is not surprising that the VISTA enhancers were selected for validation in transgene animal models due to their ultra-conservation [28] and thus are mainly involved in embryonic development [103, 104] Therefore, as in the case of the human genome [50], dePCRM2 is able to partition the peak-covered genome regions into a functional set, i.e., the CRMCs, and a non-functional set, i.e., the non-CRMCs.

As we indicated earlier, there are still 20.1% of genome regions that are not covered by the extended peaks. To see whether the non-exonic sequences in these peak-uncovered regions contain functional elements such as CRMs, we plotted the distribution of the phyloP scores of their genomic positions. The proportion of neutrality (0.83) of these positions is in between those of the peak-covered regions (0.78) and those of the non-CRMCs (0.89) (Fig. 4A), suggesting that they might contain functional elements, albeit with a lower density than that in the peak-covered regions. Based on the difference in the proportion of neutralities of the peak-covered and peak-uncovered regions as well as that of the non-CRMCs, we estimate that proportion of CRMC positions in the peak-uncovered regions is about [(1–0.83)-(1–0.89)]/[(1–0.78)-(1–0.89)] = 54.55% that of CRMC positions in the peak-covered regions.

As expected, the kept-original positions as well as the kept-extended positions have almost the same phyloP score distributions as the CRMCs (Fig. 4B), indicating that they all are under strongly evolutionary constraints. In contrast, the abandoned-original peak positions as well as the abandoned-extended positions have an almost identical phyloP score distributions to that of the non-CRMCs (Fig. 4B), indicating that they all are largely selectively natural or nearly so. These results strongly suggest that the kept extended positions are likely functional, while the abandoned-original positions are unlikely functional. This results confirm our earlier conclusion that originally called binding peaks cannot be equivalent to CRMs, and appropriate extension of the originally called short binding peaks can greatly increase the power of available datasets for predicting CRMs and constituent TFBSs in genomes [50].

### Higher-scoring CRMs are more likely under evolutionary constraints

To investigate the relationship between the evolutionary behaviors of the CRMCs and their ${S}_{CRM}$ scores, we plotted the distribution of the phyloP score of subsets of CRMCs with ${S}_{CRM}$ scores in nonoverlapping intervals. As shown in Fig. 4C and D, with the increase in the ${S}_{CRM}$ scores, the proportion of neutrality of the corresponding CRMs first drops rapidly and then enters a gradually decreasing phase. Thus, CRMCs with higher ${S}_{CRM}$ scores are more likely under evolutionary constraints, indicating that the ${S}_{CRM}$ score captures the evolutionary behavior of a CRM. Interestingly, even the CRMCs with scores in the lowest interval [0, 1) have a lower proportion of neutrality than that of the non-CRMCs (0.87 vs 0.89) (Fig. 4C and D), suggesting that even these lowest scoring CRMCs that tend to be short (Fig. 3F) are under stronger evolution constraints than the non-CRMCs, and thus are likely functional.

Next, we examined the phyloP scores for the CRMs predicted at different ${S}_{CRM}$ score cutoffs α (or *p*-values). As shown in Fig. 4E and F, with the increase in the ${S}_{CRM}$ score cutoff α, the proportion of neutrality of the predicted CRMs decreases gradually, suggesting again that the ${S}_{CRM}$ score captures the evolutionary behavior of the CRMCs. As indicated earlier, even at the lowest ${S}_{CRM}$ cutoff (α = 0), the predicted CRMs (i.e., all the CRMCs) have smaller neutral composition than that of the non-CRMCs, suggesting that at least most of the CRMC are functional, and the higher the ${S}_{CRM}$ score of a CRM, the more likely it is evolutionarily constrained, and thus the more likely it is functional.

### Predicted CRMs are supported by independent experimental data

**Fig. 5:**
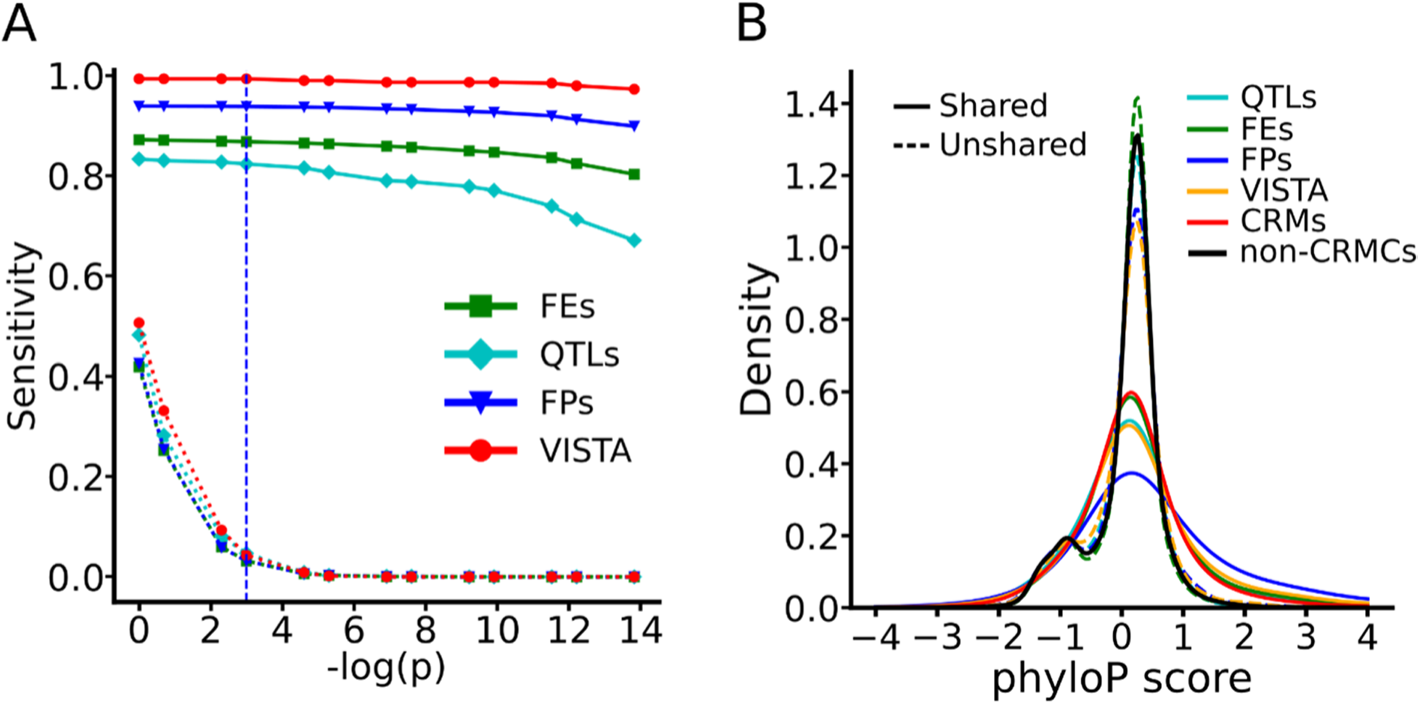
Validation of the predicted CRMs by VISTA enhancers, FANTOM promoters (FPs), FANTOM enhancers (FEs) and QTLs. **A** Sensitivity (recall rate) of the predicted CRMs or the control sequences as a function of *p*-value cutoff for recalling each set of the experimentally determined elements. The dashed vertical line indicates the *p*-value cutoff of 0.05. The sensitivity of the CRMs predicted at all the indicated *p*-value cutoffs are significantly higher (*p* < 2.2 × 10^–302^, χ^2^ test) than the control sequences for recalling each set of the experimentally determined elements. **B** Distributions of phyloP scores of the shared and unshared nucleotide positions of the elements in each set of the experimentally determined elements, in comparison with those of the predicted CRMs at *p* ≤ 0.05 and of the non-CRMCs. The difference between the distributions of shared and unshared positions in each set of the experimentally determined elements is significant, *p* < 2.2 × 10^–302^ (K-S test). Note that there are only three unrecalled VISTA enhancers

We next evaluated the sensitivity (recall rate) of our CRMs predicted at different *p*-values for recalling four types of experimentally determined CRM-related elements, including 620 mouse enhancers documented in the VISTA database [60], 163,311 mouse promoters and 49,385 mouse enhancers determined by the FANTOM project [105, 106], and 2,208 QTLs documented in the Mouse Genome Informatics (MGI) databases [107]. Interestingly, most of these experimentally determined elements are located in the peak-covered genome regions, including 579 (93.4%) VISTA enhancers, 163,311 (99.1%) FANTOM promoters and 49,385 FANTOM enhancers (99.2%) [108], with the exception for QTLs with only 1,023 (46.3%) being located in the peak-covered regions. If a predicted CRM and an element overlaps each other by at least 50% of the length of the shorter one, we say that the CRM recovers the element. As show in Fig. 5A, with the increase in the p value (decrease in -log(p)) cutoff, the sensitivity increases rapidly and saturates at a *p*-value cutoff 0.05 (α ≥ 65) to 99.3%, 93.8%, 86.8%, 82.3% for recovering the VISTA enhancers, FANTOM promoters, FNATOM enhancers and QTLs, respectively. Thus, the VISTA enhancers are largely a subset of our CRMCs. In contrast, the control sequences with the matched number and lengths of the predicted CRMs at different *p*-value cutoffs only recall an expected proportions of the elements by chance (*p* < 2.2 × 10^–302^, χ^2^ test) (Fig. 5A). Figures S2A ~ S2D show examples of the predicted CRMs that recover these four different types of experimentally determined elements.

The varying range of sensitivity from 82.3% for QTLs to 99.3% for VISTA enhancers might reflect the varying reliability of methods used to characterize these four types of elements. For example, VISTA enhancers and FANTOM promoters were determined by highly reliable transgene animal models [60] and CAGE methods [109], respectively, and our predicted CRMs achieve very high sensitivity to recall them. On the other hand, FANTOM enhancers and QTLs were determined by less reliable eRNA quantification [108] and association studies, respectively, and our predicted CRMs achieve relatively low sensitivity to recall them.

To find out whether our predicted CRMs missed these unrecalled elements, or they are simply false positives due to the limitations of experimental methods used to characterize them, we compared the phyloP scores of the recalled and unrecalled elements. As shown in Fig. 5B, for all the four types of elements, the recalled elements (solid lines) tend to be under strongly evolutionary constrains like our predicted CRMs, thus are likely functional. In contrast, the unrecalled elements (dashed lines) are largely selective neutral like our predicted non-CRMCs, thus are likely false positives produced by the methods used to characterize them. Based these results, we estimated an FDR of 0.7% (100%-99.3%), 6.2% (100%-93.8%), 13.2% (100%-86.8%) and 17.7% (100%-82.3%) in VISTA enhancers, FANTOM promoters, FANTOM enhancers and QTLs, respectively.

### Most of predicted CRMs might be in correct lengths

**Fig. 6:**
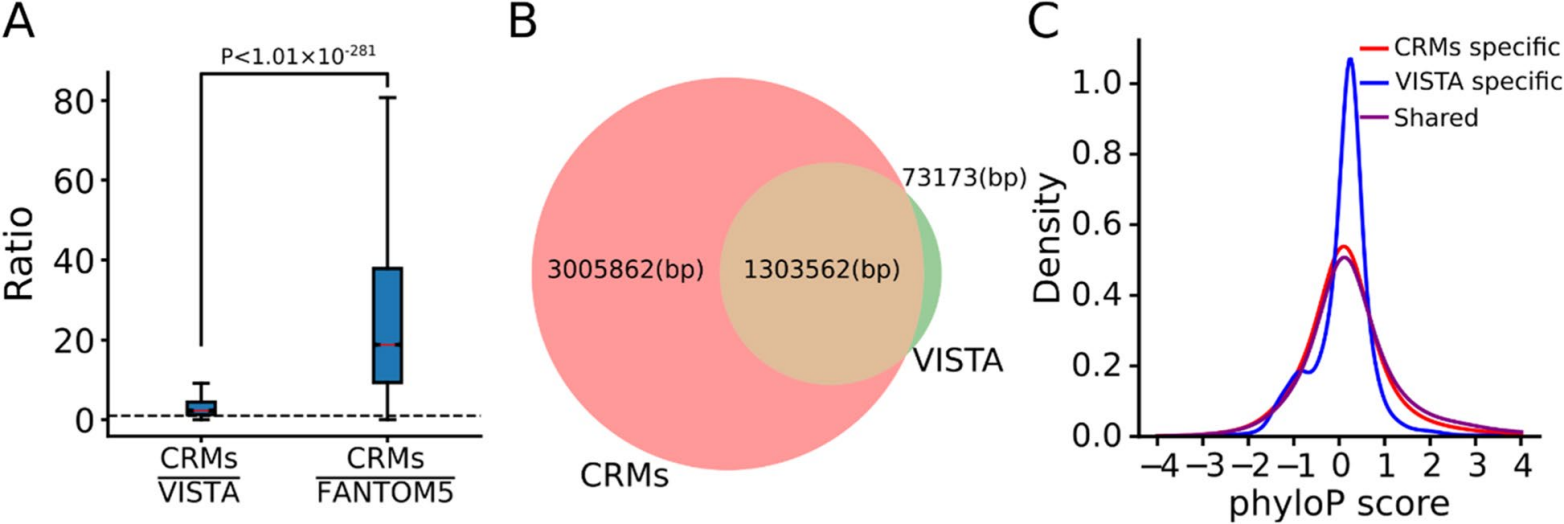
dePCRM2 might correctly predict the lengths of most CRMs. **A** Boxplots of the ratio of the length of a recalling CRM over that of its recalled VISTA enhancers and FANTOM5 enhancers. The p value was calculated using the Mann–Whitney U test. **B** Venn diagram showing the number of nucleotide positions shared by recalling CRMs and recalled VISTA enhancers, and the number of positions specific to the recalling CRMs and the recalled VISTA enhancers. **C** Distributions of phyloP scores of the positions shared by the recalling CRMs and the recalled VISTA enhancers, and of the positions specific to the recalling CRMs and to the recalled VISTA enhancers. The difference between the distributions of the shared and VISTA specific positions is significantly different, *p* < 2.2 × 10^–302^, K-S test

Correct characterization of the lengths of CRMs is notoriously difficult both experimentally and computationally, because even short components of a long CRM might still be at least partially functional in transgene animal models [28], and because functionally related independent enhancers may cluster with each other to form super-enhancers [110, 111], or locus control regions (LCRs) [112]. Although VISTA enhancers are by no means a gold standard set of CRMs with correctly characterized lengths [60], they are the only available set of validated enhancers in mouse. As we indicated earlier, our CRMs predicted at *p*-value cutoff 0.05 recall 575 (99.3%) of the 579 VISTA enhancers in the peak-covered genome regions (Fig. 5A), we thus ask whether the recalling CRMs have a length matching the recalled VISTA enhancers. To this end, we computed the ratio of the length of a recalling CRM over that of its recalled VISTA enhancer. As shown in Fig. 6A, the recalling CRMs are on average twice as long as the recalled VISTA enhancers. To see whether we over-predict the lengths of the recalling CRMs or the recalled VISTA enhancers are only shorter functional components of long enhancers, we compared phyloP scores of the 1,303,562 bp positions shared by the recalling CRMs and the recalled VISTA enhancers, with those of the 3,005,862 bp (69.75%) and 73173 bp (5.31%) positions specific to the recalling CRMs and the recalled VISTA enhancers (Fig. 6B). As expected, like our predicted CRMC positions (Fig. 4A), positions shared by the CRMs and the VISTA enhancers tend to be under strongly evolutionary constraints (Fig. 6C). Moreover, the CRM specific positions (69.75%) also tend to be under strongly evolutionary constraints (Fig. 6C) as expected, suggesting that the positions in recalling CRMs that the recalled VISTA enhancers lack might be functional. In contrast, like our predicted non-CRMCs (Fig. 4A), the VISTA enhancer specific positions (5.31%) are largely selectively neutral (Fig. 6C), suggesting that the positions in the recalled VISTA enhancers that the recalling CRMs lack might not be functional. Therefore, although the recalled VISTA enhancers are only half as long as the recalling CRMs, they might contain non-enhancer sequences that comprise 5.31% of the total length of the recalled VISTA enhancers. On the other hand, we noted that 38 (6.6%) VISTA enhancers were recalled by multiple short CRMCs, suggesting that some of short CRMCs are indeed only components of a long CRMC, whose full-length forms remain to be predicted when more TF ChIP-seq data are available in the future.

We also compared the lengths of the recalling CRMs and their recalled FANTOM5 enhancers. As shown in Fig. 6A, the recalling CRMs (median length 4,233 bp) are about 14.7 times as long as the recalled FANTOM enhancers (median length 288 bp). Moreover, 34.8% of the recalled FANTOM enhancers were located in the same CRMs. Thus, FANTOM enhancers tend to be short components of long CRMs. Taken together, these results strongly suggest that although some of our CRMCs might be short components of long CRMs, the vast majority of the CRMs predicted *p*-value cutoff of 0.05 might be in correct full length, while many VISTA enhancers and most FANTOM enhancers might be only a component of otherwise long enhancers.

### Our predicted CRMs and constituent TFBSs are more accurate and complete than existing predictions

**Fig. 7:**
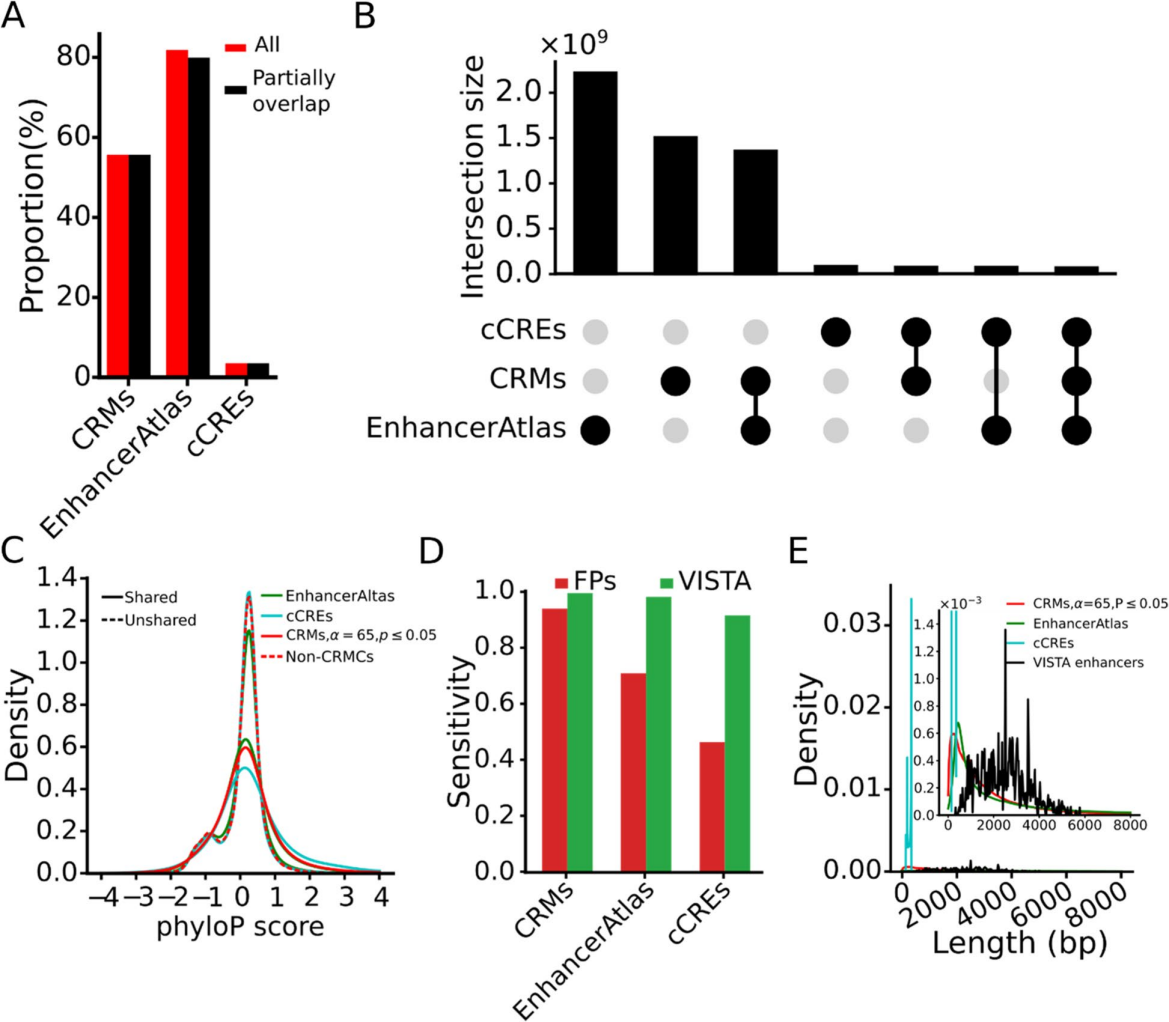
Comparison of our CRMs (*p*-value < 0.05) with the cCREs and the EnhancerAtlas enhancers. **A** Percentage of the genome covered by all the sequences of the CRMs, the cCREs and the EnhancerAtlas enhancers (All), and by the sequences in the CRMs, the cCREs and the EnhancerAtlas enhancers, which at least partially overlap the peak-covered genome regions (Partially overlap). **B** Upset plot showing numbers of nucleotide positions shared and unshared among the sequences in the three sets of predicted CRMs. **C** Distributions of phyloP scores of nucleotide positions of the cCREs and the EnhancerAtlas enhancers that are shared and unshared with our CRMs (*p*-value ≤ 0.05). **D** Comparison of sensitivity of the three sets of predicted CRMs for recalling FANTOM promoters and Vista enhancers. **E** Distributions of lengths of the CRMs, EnhancerAtlas enhancers and cCREs. The inset is a zooming-in view of the indicated range of the vertical axis

We further evaluated our 798,257 CRMs predicted at *p*-value ≤ 0.05 (${S}_{CRM}\ge 65$) with two sets of predicted mouse enhancers, including 339,815 cCREs predicted recently by the ENCODE phase 3 consortium [30] and 519,386 enhancers from the EnhancerAtlas database [42]. As shown in Fig. 7A, these three sets of predicted CRMs containing highly varying numbers of elements cover highly varying portions of the genomes, i.e., 55.5%, 3.4% and 81.6% by our CRMs, the cCREs and the EnhancerAtlas enhancers, respectively. Since all our CRMs are located in the peak-covered genome regions, we only consider for comparison the cCREs and the EnhancerAtlas enhancers that have at least one nucleotide position overlapping the peak-covered genome regions. As shown in Fig. 7A, the vast majority of the cCREs (339,721 or 99.97%) and the EnhancerAtlas enhancers (436,504 or 84.0%) have at least one nucleotide position overlapping the peak-covered genome regions. The cCREs and EnhancerAtlas enhancers that at least partially overlap the peak-covered genome regions cover 3.4% and 81.6% of the genome (Fig. 7A). Therefore, our CRMs in the peak-covered genome regions cover a much larger proportion (55.5%) of the genome than do the cCREs (3.4%), but a much smaller proportion of the genome than do the EnhancerAtlas enhancers (81.6%).

To see whether we over-predicted the CRMs with respect to the cCREs, or under-predicted the CRMs with respect to the EnhancerAtlas, we first identified the shared and unshared genome positions among the three sets of sequence elements. As shown in Fig. 7B, most (85,075,038 bp or 92.0%) of the cCRE positions overlap our CRM positions, but they only cover 5.6% of our CRM positions, while missing 94.4% of our CRM positions because of the much shorter total lengths of the cCREs (Fig. 7A). The remaining 8.0% of the cCRE positions do not overlap our CRM positions. A total of 1,364,995,621 bp (61.3%) EnhancerAtlas enhancer positions overlap our CRM positions (Fig. 7B), covering 90.3% of our CRM positions, while missing 9.7% of our CRM positions. The remaining 39.7% of the EnhancerAtlas enhancer positions do not overlap our CRMs.

We then compared the phyloP scores of the cCRE and EnhancerAtlas enhancer positions that they shared and unshared with our CRMs positions (Fig. 7B). As expected, like our CRM positions (Fig. 7C), both the cCRE and the EnhancerAtlas positions shared with our CRMs tend to be under strongly evolutionary constraints, suggesting that they are likely functional. In stark contrast, the eCRE and the EnhancerAtlas positions unshared with our CRMs are largely selectively neutral like the non-CRMCs, suggesting that they might be not functional, and thus are false positive predictions. These results suggest that the cCRE and EnhancerAtlas enhancer positions that overlap our CRMs are more likely to be functional, while those that do not overlap our CRMs are more likely to be false positives. Therefore, based on the proportion of the unshared positions, we estimate the FDRs of the cCREs and EnhancerAtlas enhancers to be about 8.0% and 39.7%, respectively.

We also compared sensitivity of our CRMs, EnhancerAtlas and cCREs for recalling FANTOM prompters and VISTA enhancers in the peak-covered genome regions. We choose the FANTOM promoters and VISTA enhancers for this validation because the high quality of the two datasets with an estimated FDR of 0.7% and 6.2%, respectively, based on their proportions of neutrality (Fig. 5B). As shown in Fig. 7D, our CRMs substantially outperform the cCREs for recalling the FANTOM promoters (93.8% vs 46.3%) and VISTA enhancers (99.3% vs 91.4%). However, this comparison might not be meaningful as the total length of our CRMs is 16 time as large as that of the cCREs. On the other hand, although the total length of our CRMs is only 68.0% that of the EnhancerAtlas enhancers, our CRMs outperform the EnhancerAtlas enhancers for recalling VISTA enhancers (99.3% vs 97.9%) and FANTOM promoters (93.8% vs 70.1%).

Finally, we compared the lengths of our CRMs with those of the cCREs and the EnhancerAtlas enhancers. As shown in Fig. 7E, the distribution of the lengths of the cCREs has a very sharp peak around 250 bp with a mean length of 272 bp, indicating that the cCREs have almost the same lengths, a possible artifact of the prediction methods. Both the distributions of the lengths of our CRMs and EnhancerAtlas enhancers are similarly strongly skewed toward right with a mean length of 1,893 and 4,285 bp, respectively. Since there is no gold standard set of full-length CRMs, we could not validate the length of our CRMs and EnhancerAtlas enhancers. However, based on the evolutionary constraints on our CRMs, most our predicted CRMs might be in full-length, while 39.7% of the EnhancerAtlas enhancers positions might be false positives as we argued earlier. Taken together, our results suggest that our CRMs might be more accurate and complete than both the cCREs and the EnhancerAtlas enhancers.

### About 64% of the mouse genome might code for CRMs

**Fig. 8:**
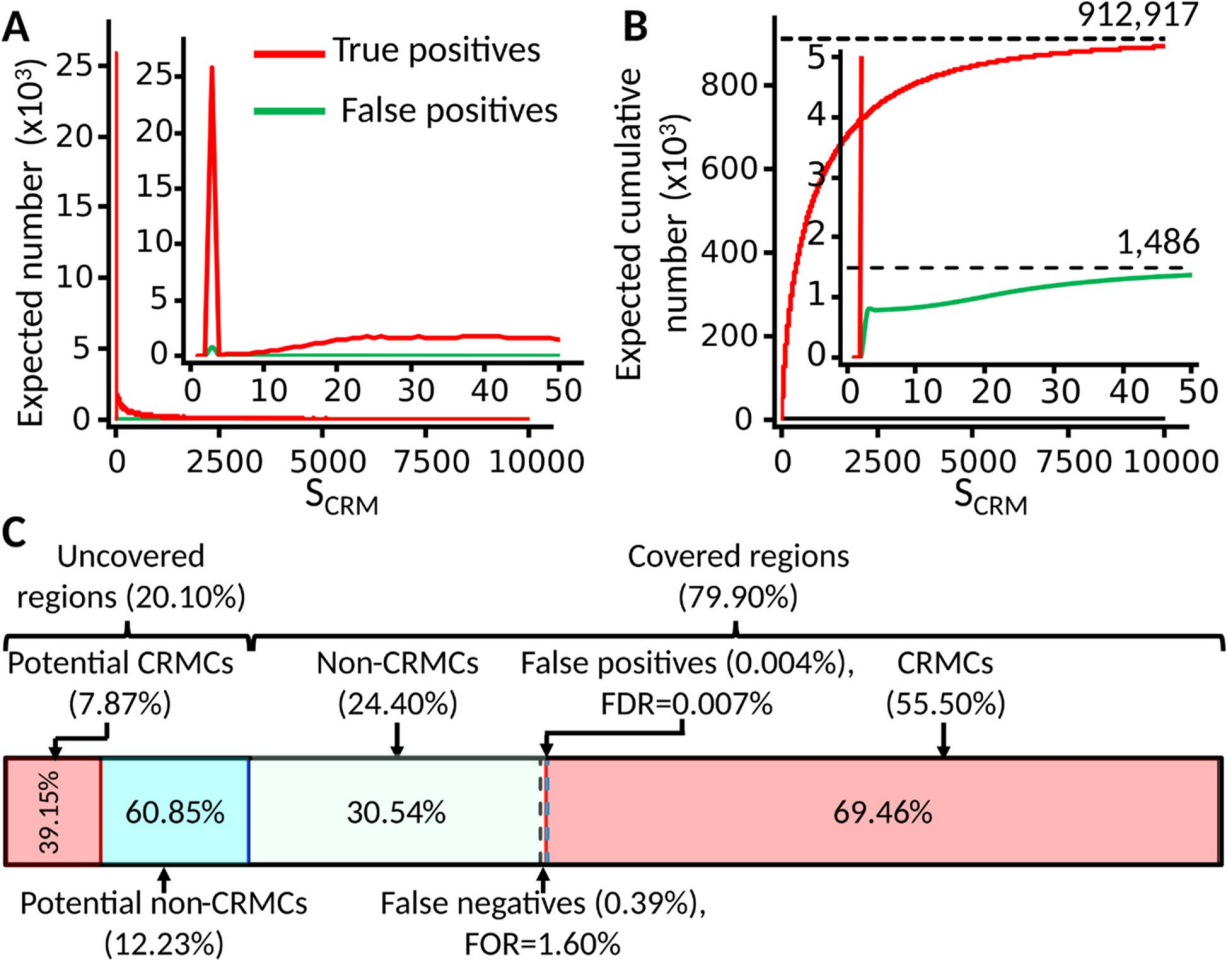
Estimation of the portion of the mouse genome encoding CRMs. **A** Expected number of true positive and false positive CRMCs in the predicted CRMCs in each one-unit interval of the ${S}_{CRM}$ scores. The inset is a blow-up view of the axes defined region. **B** Expected cumulative number of true positives and false positives with the increase in ${S}_{CRM}$ score cutoff for predicting CRMs. The inset is a blow-up view of the axes defined region. **C** Proportions of the peak-covered genome regions (79.9%) and peak-uncovered genome regions (20.1%) in the genome and estimated proportions of CRMCs in them. Percentages in the braces are the proportions of the indicated sequence types in the genome, and percentages in the boxes are the proportions of the indicated sequence types in the covered regions or in the uncovered regions

As we indicated earlier, our predicted 912,197 CRMCs make up of 55.5% of the mappable mouse genome. To estimate the FDR of the CRMCs, we took a semi-theoretic approach as we did earlier in the human genome [50]. Specifically, we calculated the expected number of true positives and false positives in the CRMCs with a ${S}_{CRM}$ score in each of non-overlapping interval based on the density of the ${S}_{CRM}$ scores of the CRMCs and the density of the ${S}_{CRM}$ scores of the Null CRMCs (Fig. 8A), yielding 910,711 (99.84%) expected true positives and 1,486 (0.16%) expected false positives in the CRMCs (Fig. 8B). Most (1,373/1,486 = 92.40%) of the 1,486 expected false positive CRMCs have a low ${S}_{CRM}$ score < 50 (insets in Fig. 8A and B) with a mean length of 64 bp, comprising 0.004% (1,486*64 bp /2,725,521,370 bp) of the mappable genome and 0.007% (0.004/55.5) of the total length of the CRMCs, i.e., an FDR of 0.007% for the CRMC positions (Fig. 8C). Thus, our predicted true CRMCs would comprise 55.5%-0.004% = 55.496% of the genome. On the other hand, as the CRMCs miss 0.7% of VISTA enhancers in the peak-covered regions [the point at -log (*p*) = 0 in Fig. 5A], we assume the FNR of predicting CRMC positions to be about 0.7%. We estimate false negative CRMC positions to be 0.007*0.55496/(1–0.007) = 0.39% of the genome, which is 0.39%/24.4% = 1.60% of the total length of the non-CRMCs, meaning a false omission rate (FOR) of 1.60% for the non-CRMC positions (Fig. 8C). Hence, true CRM positions in the peak-covered regions would be 55.5%-0.004% + 0.39% = 55.89% of the genome (Fig. 8C). In addition, as we argued earlier, the CRMC density in the peak-uncovered 20.10% genome regions is about 54.55% of that in the peak-covered genome regions, CRMCs in the uncovered regions would be about 0.201*0.5589*0.5455/0.779 = 7.87% of the genome (Fig. 8C). Taken together, we estimated about 55.89% + 7.87% = 63.76% of the genome to code for CRMs, for which we have predicted 55.89/63.76 = 87.66%. Moreover, as we predicted about 42.9% of CRMs to be made up of TFBSs (Fig. 3D), we estimated about 0.429*63.76% = 27.35% of the genome to encode TFBSs. Furthermore, assuming a mean length 1,893 bp for CRMs (the mean length of our predicted CRMs at *p*-value $\le$ 0.05), and a mean length of 17 bp for TFBS islands, we estimated that the mouse genome would encode about 918,010 CRMs (2,725,521,370 × 0.6376/1,893) and 43,848,829 non-overlapping TFBS islands (2,725,521,370 × 0.2735/17).

## Discussion

In this study, using the dePCRM2 pipeline [50], we predicted an unprecedented comprehensives map of 0.91 M CRMCs and 38.55 M constituent TFBS islands in 79.9% of the mouse mappable genome covered by 1,000 bp binding peaks in 8,884 ChIP-seq datasets for 696 TFs in 435 mouse cell line/tissue/organ types. Many features of the predicted CRMCs and TFBSs in the mouse genome are reminiscent of those of our earlier predicted CRMCs and TFBSs in the human genome [50]. First, the number of predicted UMs in both genomes are very close (238 vs 210), reflecting the fact that both genomes encode highly conserved sets of TF families [66, 113]. Second, most of the UMs in both genomes match known TF motif families, and most known motif families are matched by the UMs in both genomes. Third, the mouse CRMCs consist of 55.5% of the mouse genome, while the human CRMCs make up of 44.0% of the human genome [50]. The higher genome coverage of the mouse CRMCs are clearly due to a larger number (9,060 vs 6,092) of available TF ChIP-seq datasets covering a higher proportion (79.9% vs 77.5%) of the mouse genome were used. Fourth, peak-uncovered regions in both genome may still contain CRMs albeit at a lower density than the peak-covered regions according to their evolutionary profiles (Fig. 4A) [50]. To predict CRMs and constituent TFBSs in these peak-uncovered regions in both genomes, more TF ChIP-seq data, particularly, for new TFs in new cell/tissues of human and mouse are needed to cover these currently peak-uncovered regions. We expect that with more TF ChIP-seq datasets available in both the human and mouse cell/tissue types, the peak-covered genome regions would increase and eventually become saturated [50, 61]. Fifth, we estimated that about 63.8% (Fig. 8C) and 55.4% [50] of the mouse and human genomes might encode CRMs, and TFBSs make up of about 40% of the lengths of the CRMs in both genomes. Therefore, CRMs might be more prevalent than originally thought in both the mouse and human genomes. However, they might not be as prevalent (81% and 59% in the mouse and human genomes, respectively) (Fig. 7C) [50] as the EnhancerAtlas database documented [42].

Sixth, the predicted CRMCs in both genomes are more likely subject to evolutionary constraints than the predicted non-CRMCs that are largely selectively neutral or nearly so. Hence, the CRMCs are likely *cis*-regulatory, while the non-CRMCs are unlikely *cis*-regulatory. Seventh, the predicted CRMCs in both genomes achieve very high sensitivity for recalling CRM-related elements determined by highly reliable methods, such as the VISTA enhancers and FANTOM promoters. Eighth, recalling CRMs in both genomes are about twice as long as the recalled VISTA enhancers, and the unshared positions in the recalling CRMs are subject to strong evolutionary constrains, while unshared positions in the recalled VISTA enhancers are not. Therefore, most of the predicted CRMCs in both genomes are likely in correct full-lengths, particularly, those with higher ${S}_{CRM}$ scores and lower *p*-values, while some VISTA enhancers might be only components of long CRMs, but still are at least partially functional [3, 114]. However, a small portion of the predicted CRMCs in both genomes might be short components of long CRMs, particularly, those with low ${S}_{CRM}$ scores and higher *p*-values. Clearly, more TF ChIP-seq data are needed to cover the relevant genome regions to predict them in full-lengths.

Nineth, the predicted CRMCs in both genomes are substantially more complete and more accurate than those predicted by other state-of-the-art methods measured by evolutionary constraints (Fig. 7C) and sensitivity for recalling experimentally determined VISTA enhancers and FANTOM5 promoters. Thus, dePCRM2 is a powerful and robust method for de novo prediction of CRMs and TFBSs in large mammal genomes by integrating a very large number of TF ChIP-seq datasets. Tenth, we predicted 42.1% and 30.5% of the CRMC positions in the human and mouse genomes, respectively, based on the extended parts of the sequences. Therefore, extending the lengths of most of originally called peaks to 1,000 bp could substantially increase the power of the available datasets. On the other hand, we predicted 37.8% and 24.8% of originally called peak positions in the human and mouse genomes, respectively, to be non-CRMCs. Thus, the originally called binding peaks might not be equivalent to parts of CRMs. These results reflect the noisy nature of TF ChIP-seq data and the fact that although TFBSs of a ChIP-ed TF are typically located in the middle of called peaks, those of its cooperative TFs can reside anywhere along the flanking regions of the peak within the host CRM [50, 62].

Finally, although the functional states (TF binding or non-TF-binding) of some CRMs in a cell/tissue type can be predicted based on the overlaps of the CRMCs and TF binding peaks available in the cell type [50], functional states of most of the predicted CRMCs in most cell types in both organisms are currently agnostic due to the limited availability of TF ChIP-seq data in most cell types. Fortunately, it has been shown that when the locus of a CRM is accurately anchored by the bindings of key TFs, few epigenetic marks can be an accurate predictor of the functional state of the CRM [40, 48, 49, 53, 115]. Thus, the second step of our proposed two-step approach is to predict the functional states of all the predicted CRMs in any cell type in an organism using a minimal set of epigenetic marks collected from the very cell type.

With the availability in the future of even more TF ChIP-seq datasets for more diverse TFs in more diverse cell/tissue types of humans and mice, as well as of other important model organisms such as *Caenorhabditis elegans, Drosophila melanogaster* and *Arabidopsis thaliana,* we are hopeful to predict even more accurate and complete maps of CRMs and constituent TFBSs in all these genomes. These maps will facilitate characterizing functional states and target genes of the CRMs in various cell/tissue types of the organisms, and elucidating the rules of organization and evolution of CRMs and constituent TFBSs at a genome scale.

## Methods

### Datasets

We downloaded the.narrowPeak BED files for 9,060 mouse TF ChIP-seq datasets (Table S1) from the Cistrome database [116]. The binding peaks in each dataset were uniformly called by a pipeline based on the MACS program [117], and each binding peak was assigned with a score (the 5^th^ column in the.narrowPeak BED files) that measured enrichment of the ChIP-seq reads count in the peak relative to the influence of local biases [116]. We filtered out low-quality peaks with an enrichment score less than 20 in each dataset. We discarded filtered datasets with fewer than 20 binding peaks, resulting in 8,884 datasets used in the subsequent predictions. For each called binding peak in each dataset, we extracted a 1,000 bp peak centered on the middle of the peak. We did so, because almost all known mammal enhancers with a mean length about 2,400 bp [60] were longer than the mean length (315pb) of the called binding peaks and TFBSs are scattered along the entire lengths of enhancers [62, 118–120].

To validate our predictions, we downloaded 620 mouse enhancers from the VISTA Enhancer database [60], a total of 49,385 mouse enhancers and 163,311 mouse promoters from the FANTOM5 data portal [105, 106], and 2,208 QTLs from Mouse Genome Informatics (MGI) databases [107]. Two compared our predictions with existing methods, we downloaded 339,815 mouse cCREs [30] and 519,386 mouse EnhancerAtlas enhancers [42] from the respective websites.

### Measurement of the overlap of binding peaks between two different datasets

We calculate an overlap score ${S}_{0}\left({d}_{i},{d}_{j}\right)$ of binding peaks between each pair of datasets ${d}_{i}$ and ${d}_{j}$, defined as,

1 $${S}_{0}\left({d}_{i},{d}_{j}\right)=\frac{1}{2}\times \left(\frac{o\left({d}_{i}+{d}_{j}\right)}{\left|{d}_{i}\right|}+\frac{o\left({d}_{i}+{d}_{j}\right)}{\left|{d}_{j}\right|}\right)$$

## Prediction of CRMs and constituent TFBSs

To predict CRMs and constituent TFBSs in the mouse genome, we applied the dePCRM2 pipeline [50] to the datasets containing 1,000 bp peaks. DePCRM2 predicts CRMs and constituent TFBSs by identifying repeatedly cooccurring TFBSs of cooperative TFs in the 1,000 bp binding peaks in all the collected ChIP-seq datasets for various TFs in different cell/tissue types of the organism [50, 61]. This design of dePCRM2 was based on the observation that most cooperative TFs are often reused in various cell/tissue types at different developmental stages and/or under different homeostasis conditions [3]. Briefly, we first identify motifs using ProSampler [62]. Secondly, we find the highly frequently co-occurring motifs pairs (CPs) in each dataset by computing a co-occurring score, defined as

2 $${S}_{c}\left({M}_{i}\left(i\right),{M}_{j}\left(j\right)\right)=\frac{o\left({M}_{d}\left(i\right),{M}_{d}\left(j\right)\right)}{max\left\{\left|{M}_{d}\left(i\right)\right|,\left|{M}_{d}\left(i\right)\right|\right\}}$$

where |*M*_*d*_(*i*)| and |*M*_*d*_ (*j*)| are the number of binding peaks containing TFBSs of motifs *M*_*d*_(*i*) and *M*_*d*_ (*j*), respectively; and $o\left({M}_{d}\left(i\right),{M}_{d}\left(j\right)\right)$ the number of binding peaks containing TFBSs of both the motifs in *d*. Thirdly, we cluster highly similar motifs in CPs across all the datasets, and find a representative motif in each resulting motif cluster as a unique motif (UM) using ProSampler [62]. Fourthly, we construct an interaction network $N$ to model cooccurrence patterns of the UMs and interactions between their cognate TFs. In $N$, the nodes are the UMs that are fully connected, and the edge between UMs ${U}_{i}$ and ${U}_{j}$ is weighted using an interaction score, defined as,

3 $${S}_{INTER}\left({U}_{i},{U}_{j}\right)=\frac{1}{|D({U}_{i}{,U}_{j})|}\sum_{d\in D({U}_{i},{U}_{j})}(\frac{1}{|d\left({U}_{i}\right)|}+\frac{1}{|d\left({U}_{j}\right)|}){\sum }_{s\in S(d\left({U}_{i}\right),(d({U}_{j}))}\frac{150}{r(s)},$$

where ${D(U}_{i}{, U}_{j})$ is the datasets in which TFBSs of both ${U}_{i}$ and ${U}_{j}$ occur, $d({U}_{k})$ the subset of dataset $d$, containing at least one TFBS of ${U}_{k}$, $S(d\left({U}_{i}\right),(d({U}_{j}))$ the subset of $d$ containing TFBSs of both ${U}_{i}$ and ${U}_{j}$, and $r(s)$ the shortest distance between any TFBS of ${U}_{i}$ and any TFBS of ${U}_{j }$ in a sequence $s\in S(d\left({U}_{i}\right),(d({U}_{j}))$. Fifthly, we connect two adjacent TFBSs of the UMs if their distance *d* ≤ 300 bp and predict the connected segment to be a CRM candidate (CRMC) and at the same time, we predict a sequence in the peak-covered regions that cannot be connected to be a non-CRMC. In this way, we partition the peak-covered genome regions in two exclusive sets, i.e., the CRMCs and the non-CRMCs. Sixthly, we evaluate each CRMC containing $n$ TFBSs, (${b}_{1},{b}_{2}\cdots ,{b}_{n}),$ by computing a CRM score, defined as,

4 $${S}_{CRM}\left({b}_{1},{b}_{2}\cdots ,{b}_{n}\right)=\frac{2}{n-1}\sum_{i=1}^{n}\sum_{j>i}{S}_{INTER}\left[U\left({b}_{i}\right),U({b}_{j})\right]\left[S\left({b}_{i}\right)+S({b}_{j})\right]$$

where $U\left({b}_{k}\right)$ is the UM of TFBS ${b}_{k}$, ${S}_{INTER}\left[U\left({b}_{i}\right),U({b}_{j})\right]$ the interaction score between $U\left({b}_{i}\right)$ and $U\left({b}_{j}\right)$ in $N$, $S({b}_{k}$) the binding score of ${b}_{k}$ based on the position weight matrix (PWM) of $U\left({b}_{k}\right)$ [121]. Only TFBSs with a positive score are considered. Seventh, we evaluate the statistical significance of each predicted CRMC. To do so, we first generate a Null CRMC set with matched lengths and nucleotide frequencies of the CRMCs using a third order Markov chain model [62], and a random interaction network *N*ʹ generated by randomly shuffling the weights in $N$. Then, we compute the ${S}_{CRM}$ score for each Null CRMC using formula (4). We compute an empirical *p*-value for a CRMC with a ${S}_{CRM}=s$, defined as,

5 $$p =\frac{n(s)}{M},$$

where $n(s)$ is the number of Null CRMCs with a ${S}_{CRM}>s,$ and $M$ the total number of the CRMCs. Finally, dePCRM predicts functional states (TF-binding or non-TF-binding) in a cell/tissue type of the CRMs whose constituent TFBSs overlap binding peaks of ChIP-ed TFs in the cell/tissue type [50].

## Supplementary Information

**Additional file 1:** **Table S1.** Summary of TF ChIP-seq datasets of mouse cell/tissue types. **Table S2.** Unique motifs (UMs) matching known motifs of TFs in the Hocomoco and JASPAR databases.

**Additional file 2:** **Figure S1.** Prediction of UMs. A. Similarity graphs of member motifs in the 245 motif clusters. In each graph, a node in blue represents a member motif of the cluster, and two member motifs are connected by an edge in green if their similarity is greater than 0.8 (SPIC score). Clusters with the names in RED font are those in which a UM cannot be found. B. Logos of the 201 UMs found in the corresponding clusters. **Figure S2.** Examples of predicted CRMs that recover experimentally determined cis-regulatory sequence elements. A. A CRM (chr17:44617516-44620464) recovers a VISTA enhancer located in gene *Runx2*. B. A CRM (chr18:46916221-46916642) recovers a FANTOM5 enhancer (chr18:46916185-46916414) upstream of gene *Arl14epl*. C. A CRM (chr3:95542968-95543483) recovers a FANTOM5 promoter (chr3:95543009-95543137) located in gene *Ctss*. D. A CRM (chr16:61562958-61563095) recovers an MGI QTL (chr16:61562987-61563143) upstream of gene Epha6 and XR_38917.3. The inset is a zooming-in view of the QTL and the CRM.

## Data Availability

The predicted CRMs and constituent TFBSs in the mouse genome are available at http://cci-bioinfo.uncc.edu. The TF ChIP-seq binding peaks used in the current study are available at http://cistrome.org/db/#/
