# Supplementary material for: A map of cis-regulatory modules and constituent transcription factor binding sites in 80% of the mouse genome
Source: BMC Genomics. 2022 Oct 19;23:714. doi: 10.1186/s12864-022-08933-7 (PMC9583556; doi:10.1186/s12864-022-08933-7)
Supplement: Supplementary file 2 — Additional file 2: Figure S1. Prediction of UMs. A. Similarity graphs of member motifs in the 245 motif clusters. In each graph, a node in blue represents a member motif of the cluster, and two member motifs are connected by an edge in green if their similarity is greater than 0.8 (SPIC score). Clusters with the names in RED font are those in which a UM cannot be found. B. Logos of the 201 UMs found in the corresponding clusters. Figure S2. Examples of predicted CRMs that recover experimentally determined cis-regulatory sequence elements. A. A CRM (chr17:44617516-44620464) recovers a VISTA enhancer located in gene Runx2. B. A CRM (chr18:46916221-46916642) recovers a FANTOM5 enhancer (chr18:46916185-46916414) upstream of gene Arl14epl. C. A CRM (chr3:95542968-95543483) recovers a FANTOM5 promoter (chr3:95543009-95543137) located in gene Ctss. D. A CRM (chr16:61562958-61563095) recovers an MGI QTL (chr16:61562987-61563143) upstream of gene Epha6 and XR_38917.3. The inset is a zooming-in view of the QTL and the CRM. [file 12864_2022_8933_MOESM2_ESM.docx]

A


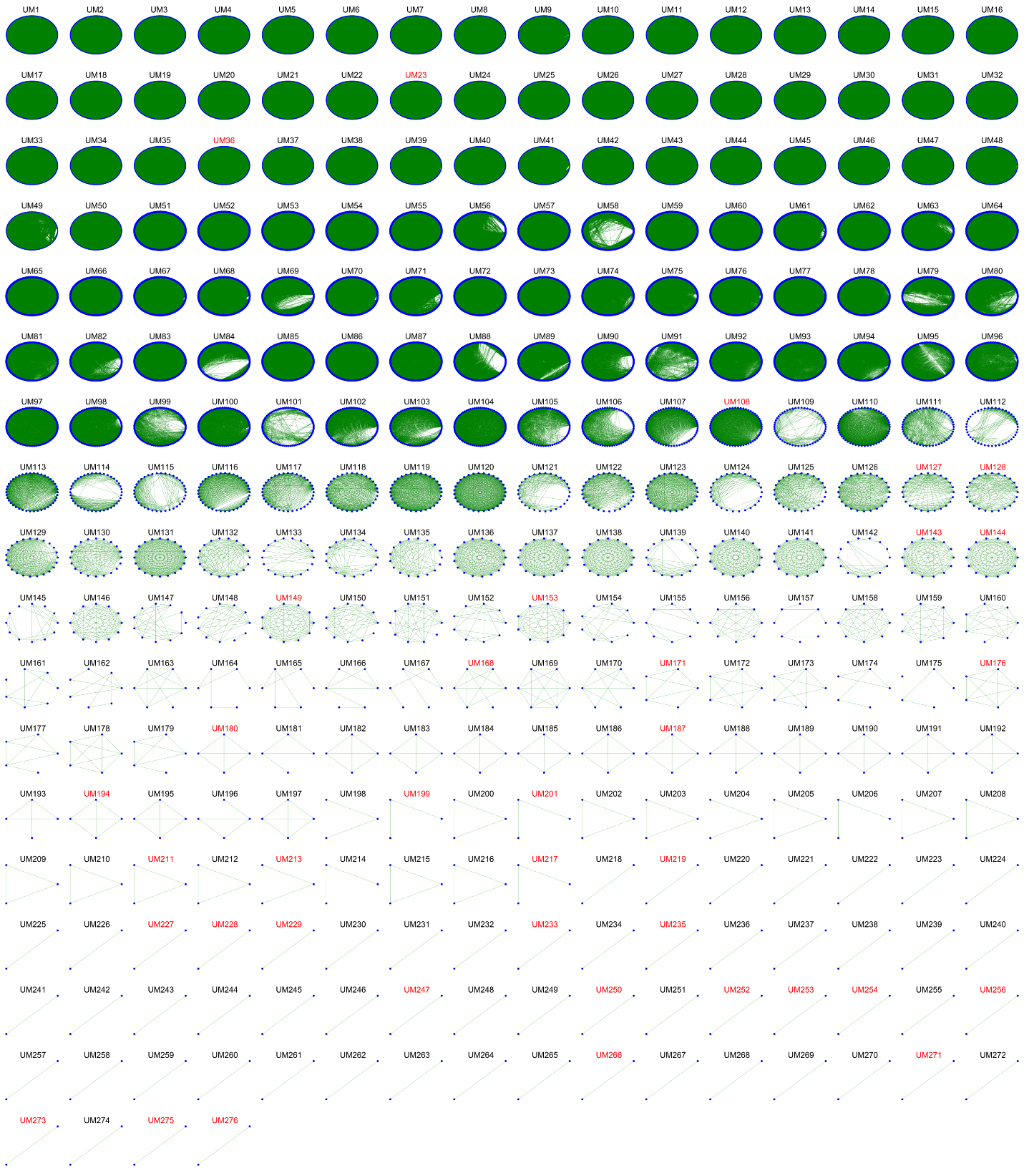


B


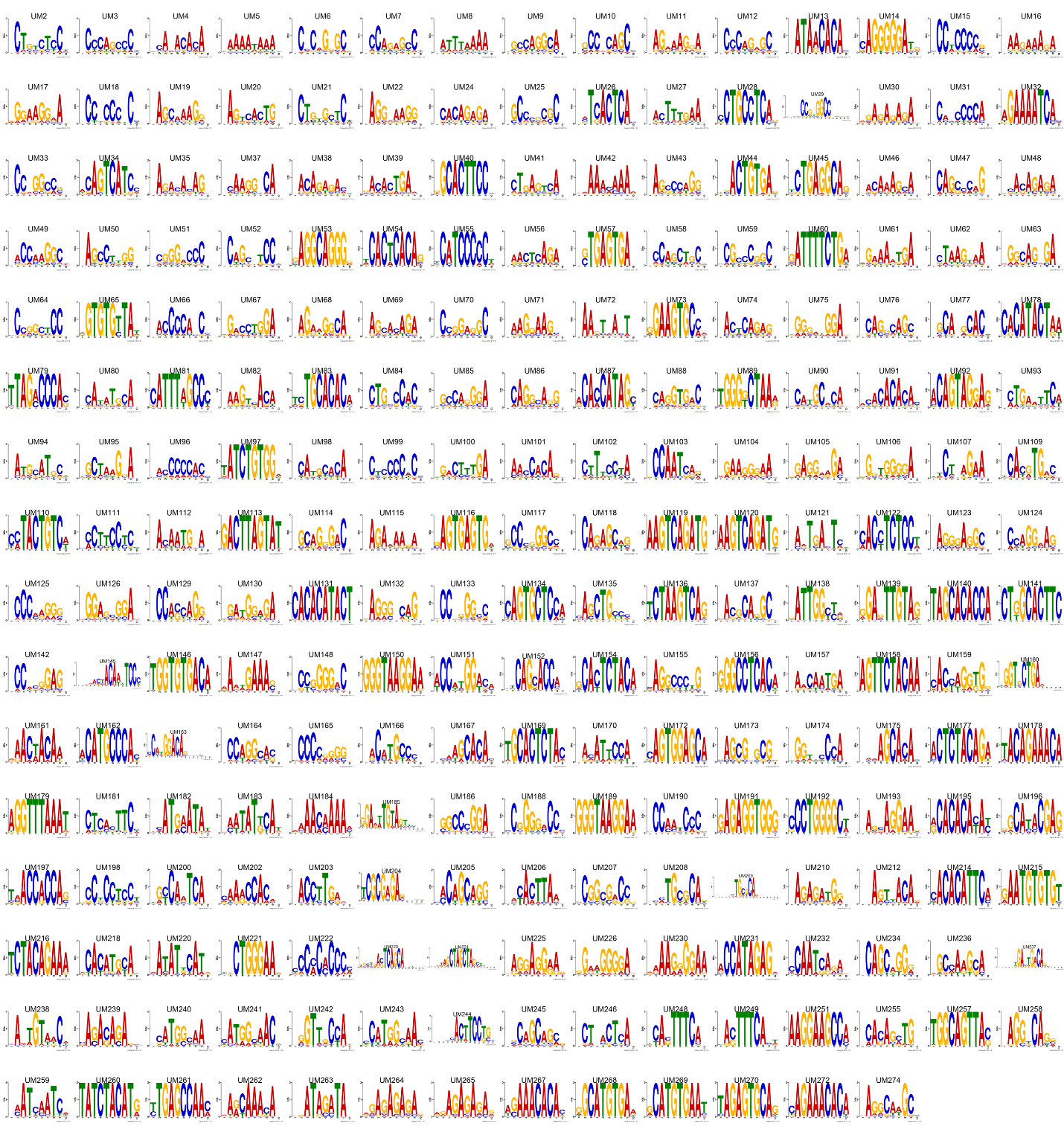


Figure S1. Prediction of UMs. A. Similarity graphs of member motifs in the 245 motif clusters. In each graph, a node in blue represents a member motif of the cluster, and two member motifs are connected by an edge in green if their similarity is greater than 0.8 (SPIC score). Clusters with the names in RED font are those in which a UM cannot be found. B. Logos of the 201 UMs found in the corresponding clusters.


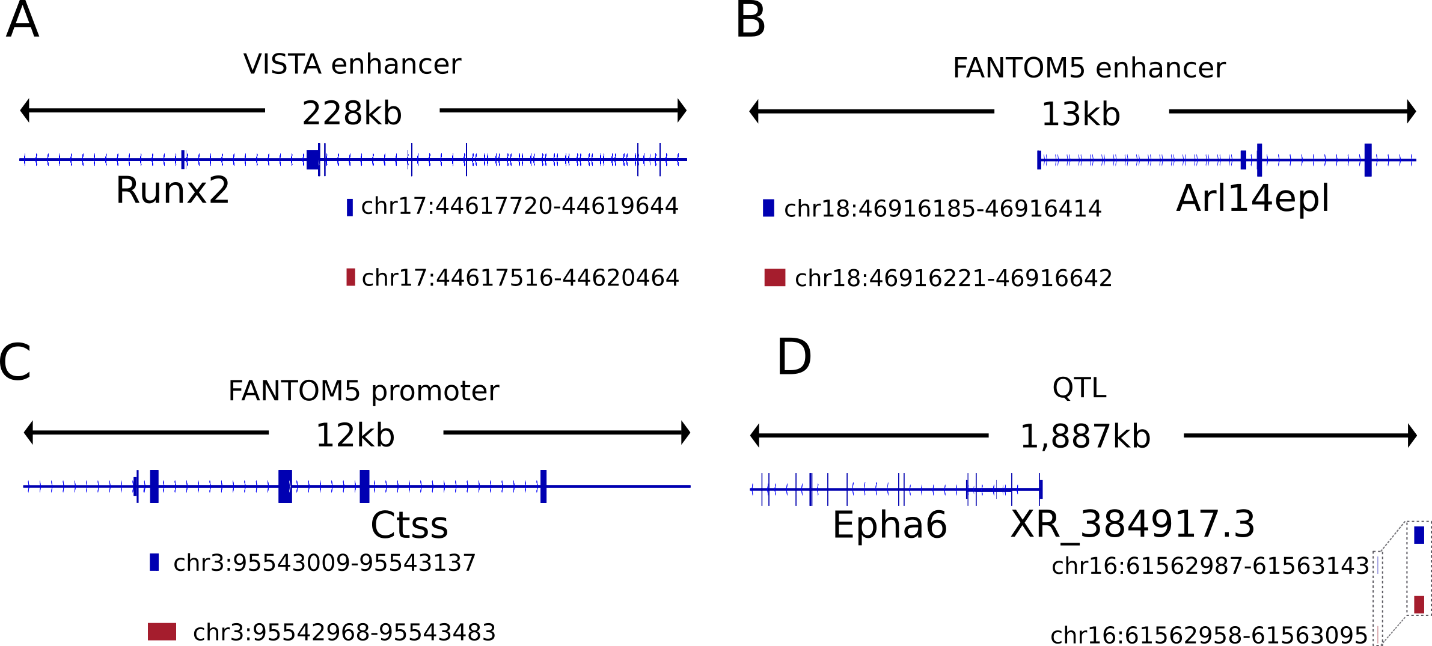


Figure S2. Examples of predicted CRMs that recover experimentally determined cis-regulatory sequence elements. A. A CRM (chr17:44617516-44620464) recovers a VISTA enhancer located in gene *Runx2*. B. A CRM (chr18:46916221-46916642) recovers a FANTOM5 enhancer (chr18:46916185-46916414) upstream of gene *Arl14epl*. C. A CRM (chr3:95542968-95543483) recovers a FANTOM5 promoter (chr3:95543009-95543137) located in gene *Ctss*. D. A CRM (chr16:61562958-61563095) recovers an MGI QTL (chr16:61562987-61563143) upstream of gene Epha6 and XR_38917.3. The inset is a zooming-in view of the QTL and the CRM.
